# Antifungal bio-coating of endotracheal tube built by overexpressing the *MCP1* gene of *Saccharomyces boulardii* and employing hydrogel as a “house” to antagonize *Candida albicans*

**DOI:** 10.1186/s40824-023-00443-1

**Published:** 2023-10-05

**Authors:** Yunyun Wei, Jianfeng Qiu, Ziqiang Han, Xuanyi Wang, Hui Zhang, Xinya Hou, Xiangwei Lv, Xiaolong Mao

**Affiliations:** 1 School of Radiology, The Second Affiliated Hospital of Shandong First Medical University, Tai’an, 271016 China; 2 School of Radiology, Shandong First Medical University and Shandong Academy of Medical Sciences, Tai’an, 271000 China; 3 Science and Technology Innovation Center, Shandong First Medical University & Shandong Academy of Medical Sciences, Jinan, 250000 China; 4 School of Laboratory Animal & Shandong Laboratory Animal Center, Shandong First Medical University & Shandong Academy of Medical Sciences, Jinan, Shandong 250000 China; 5 Department of Clinical Medicine, Shandong First Medical University and Shandong Academy of Medical Sciences, Jinan, 250000 China

**Keywords:** Medical device coating, Fungal infection, Probiotics, Antagonism, Hydrogel

## Abstract

**Background:**

For some ICU patients, an artificial airway must be established with an endotracheal tube, but *Candida albicans* can easily adhere to the tube surface and form a biofilm, leading to potentially life threatening fungal infections. Therefore, it is urgent to prevent and reduce *C. albicans* infections introduced by the endotracheal tube. However, there are few antifungal drugs effective against *C. albicans*, and each of these drugs may have adverse effects on human cells. *Saccharomyces boulardii* is regarded as an alternative strategy to inhibit the adhesion of *C. albicans*, but it is affected by environmental stress. We hypothesized that it is feasible to strengthen the antagonistic ability of *S. boulardii* via encapsulating and genetically modification.

**Methods:**

In this study, a bioactive material carrying the overexpressed *MCP1* gene of *Saccharomyces boulardii* was constructed based on one-step photo-crosslinking. This material achieved spatial growth control of *S. boulardii* by encapsulating each *S. boulardii* cell within a hydrogel pore. The bioactive material was coated on an endotracheal tube and tested for its ability to inhibit the adhesion of *C. albicans*. Additionally, the material’s antagonistic activity towards *C. albicans* was evaluated by detecting intracellular Adenosine-triphosphate content, reactive oxygen species level and the activity of antioxidative enzymes. Tissue invasion experiment was executed to further evaluate the anti-adhesion ability of *S. boulardii* bio-coating.

**Results:**

Encapsulating the overexpression of *MCP1* by *S. boulardii* in hydrogel pores enhanced the viability of probiotics in the presence of high salt and oxidation stress. When used as the coating of an endotracheal tube, the *S. boulardii* bioactive material efficiently inhibited the adhesion of *C. albicans* by impairing the activities of superoxide dismutase and catalase and disturbing mitochondrial functions. In vivo, the *S. boulardii* bioactive material coating displayed good biocompatibility and reduced the host tissue invasion and virulence of *C. albicans*.

**Conclusions:**

The integration of genetic modification and immobilization model breaks the bottleneck of previous application of microorganisms, and provides a new way to prevent fungal infections introduced by endotracheal tubes.

**Graphical Abstract:**

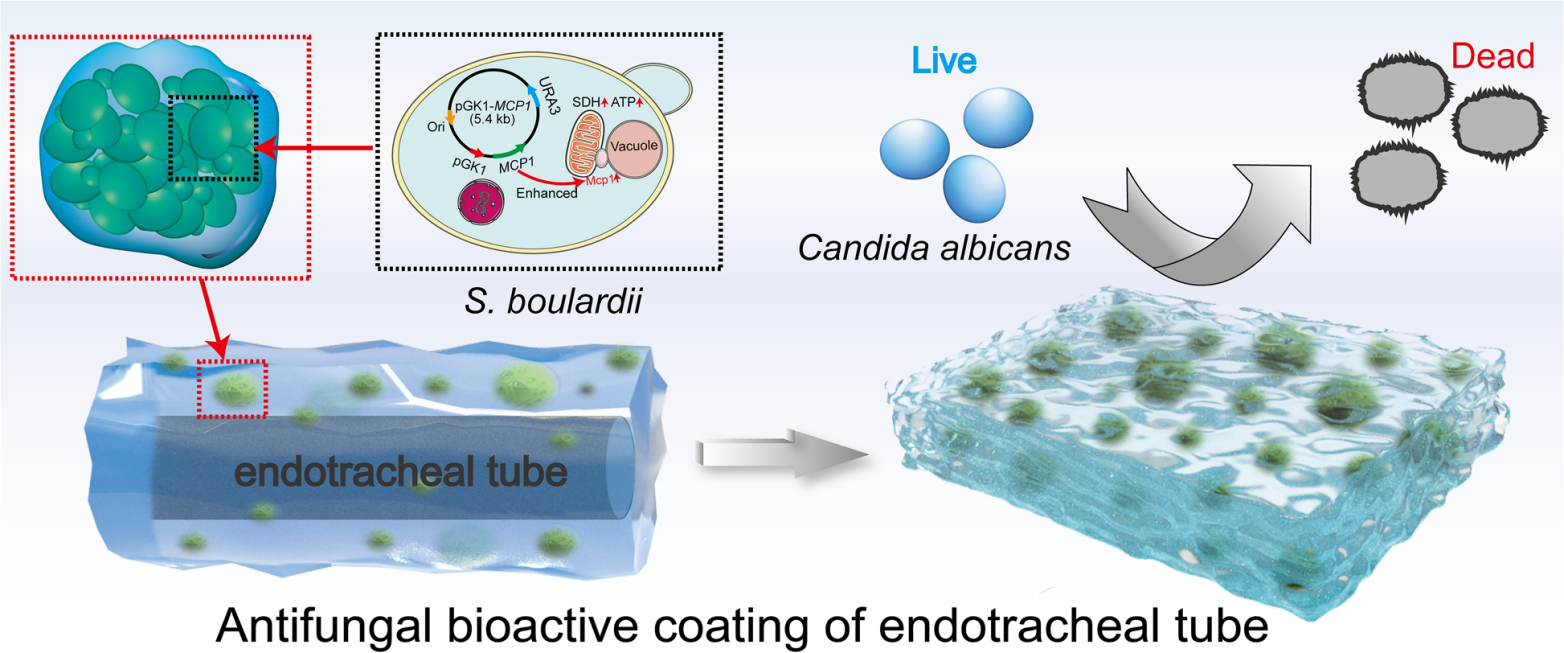

**Supplementary Information:**

The online version contains supplementary material available at 10.1186/s40824-023-00443-1.

## Background

The endotracheal tube is an essential medical device used to sustain the lives of many patients in critical condition [1]. However, retention of the endotracheal tube provides conditions for the adhesion and biofilm formation of pathogenic fungi, which potentiates biofilm-related infections, especially fungal infections [2, 3]. *Candida albicans* (*C. albicans*), a common opportunistic fungal pathogen, can easily adhere to the surface of an endotracheal tube, resulting in systemic fungal infection when the immune function of the host declines [4–6]. However, due to the excessive use of antibiotics, chemotherapy drugs, and immunosuppressants, the incidence of invasive *C. albicans* infections has increased steadily [7, 8]. It is urgent to develop an effective method to prevent the adhesion of *C. albicans* to endotracheal tubes.

Currently, many strategies are adopted to impede the adhesion of pathogenic fungi on medical devices, mainly divided into two categories: (1) placing antifungal drugs directly on the medical device and (2) giving the medical device a coating that released antifungal molecules [9]. Nevertheless, there are limited antifungal drugs, and all such drugs can damage eukaryotic cells in the host and lead to the emergence of drug-resistant strains [10]. Biotherapy, especially safe probiotic therapy, is considered a promising strategy to antagonize *C. albicans*. *Saccharomyces boulardii*, as a commercialized probiotic, serves multiple functions, such as enriching the nutritional value of fermented foods, promoting intestinal absorption, stimulating the immune system, and preventing diarrhea [11–13]. Strikingly, *S. boulardii* exerts broad antagonism properties towards pathogens. *S. boulardii* suppresses bacterial growth by outcompeting pathogens for living space, nutrients, and adhesion sites [14]. Furthermore, *S. boulardii* secretes antibacterial substances and killer toxins that are harmful to pathogens [15]. More significantly, *S. boulardii* is the only beneficial fungus currently used in humans [16, 17]. Because of its safety and antibacterial properties, *S. boulardii* is regarded as a valuable alternative therapy for preventing pathogen infections. Recent reports indicate that *S. boulardii* can inhibit the adhesion and hyphal development of *C. albicans* [18]. Our previous work also confirmed the repression of *C. albicans* adhesion by *S. boulardii* (Fig. S1), suggesting its potential to prevent *Candida* infections. However, free *S. boulardii* is difficult to colonize on the surface of medical devices, and it is easily damaged under hostile environmental stresses, such as the presence of bile salt, metal ions, and oxidative stress [19].

Genetic modification is considered as an effective strategy to enhance the stress resistance of microorganism [20]. Tang’ group constructed multiple stress-tolerant industrial *Saccharomyces cerevisiae* based on gene overexpression [21]. Huang et. al found that *Aspergillus niger* could keep alive, self-regenerative, and functional stability by tuning the inducible expression of genes [22]. Mitochondria, as centers of substance and energy metabolism, not only provide energy for eukaryotic cells through oxidative phosphorylation but also participate in numerous signal transduction and biological metabolic processes [23, 24]. Therefore, maintaining the functional stability of mitochondria is crucial for cell survival, reproduction, and growth [25]. Mcp1 (mitochondrial outer membrane protein10 complementing protein1) is a mitochondrial protein that maintains mitochondrial functions by recruiting Vps13 (which contributes to vacuolar protein sorting) [26], promotes cell growth, and restores mitochondrial respiratory chain complexes [27]. Moreover, our previous study found that deleting the *MCP1* gene impaired mitochondrial mitophagy and functions under stress conditions [28]. Thus, overexpressing *MCP1* is expected to enhance the stress resistance of cells.

An alternative method to preserve cell viability is to encapsulate microorganisms in a matrix [29, 30]. Most materials used for immobilization cannot provide suitable living conditions for microorganisms, so selecting an appropriate supporting matrix for the immobilization and growth of microorganisms is of great importance [31]. Given the favorable bio-functionality and mechanical property, hydrogel have been developed to carry cells or microorganisms in to maintain their activity and functions [32–35]. *Lactobacillus acidophilus* targeting the gastrointestinal tract was successfully trapped into hydrogels with acceptable viability by Risbo’ group [36]. Yuan et. al found that the encapsulated probiotics in composite hydrogel exhibited a high survival rate upon long-time storage [37]. Therefore, hydrogel is regarded as a promising platforms to delivery engineered living bacteria for the treatment of typical diseases. However, encapsulating *S. boulardii* in hydrogel structures with tridimensional geometries and site-specific cellular composition remains largely unexplored.

We hypothesized that it is feasible to enhance the antagonistic ability of *S. boulardii* by encapsulating it within hydrogel and genetically modification. Gelatin methacrylate (GelMA) hydrogel was used to encapsulate* S. boulardii*, because it has biocompatibility, mechanical tenability, and tunable degree of polymeric crosslinking [38, 39]. To provide a suitable living space for *S. boulardii*, Polyethylene oxide (PEO) as a porogenic agent endowed GelMA hydrogel with porosity [40]. Furthermore, we overexpressed *MCP1* gene of *S. boulardii* to maintain the functional stability of mitochondria that is important for cell viability and probiotic properties [27, 28]. The antagonistic ability of *S. boulardii* in the hydrogel was detected by mitochondrial functions assays and tissue invasion test. This design introduces a new strategy to prevent fungal infections in patients with endotracheal tubes.

## Methods

### Construction of *S. boulardii* with overexpressed *MCP1* gene and fluorescent-labeled strains

*S. boulardii* (*ura3*/*ura3*) was grown overnight in yeast extract peptone dextrose (YPD) medium (1% yeast extract, 2% tryptone, and 2% glucose) with the concentration of 80 μg/mL uridine at 30 °C with shaking at 160 rpm [41]. *S. boulardii* was centrifuged and collected in phosphate buffered saline (PBS) before use. Then, the overexpressed *MCP1* of *S. boulardii* mutant, that is, *S. boulardii* (pGK1-*MCP1*), was constructed by transformation of the pGK1-*MCP1*-*URA3* plasmid. Moreover, the *S. boulardii* (pGK1-Mcp1-*GFP*) mutant was constructed by transformation of the pGK1-*MCP1*-*GFP*-*URA3* plasmid. After the *MCP1*:*GFP:URA3* fragment was transformed into *S. boulardii* (*ura3/ura3*), the *S. boulardii* (Mcp1-*GFP-URA3*) mutant was constructed [28]. In addition, the mitochondrial outer membrane protein was observed by inverted fluorescence microscopy (NIB600, Nanjing Jiangnan Novel Optics Co., Ltd, Nanjing, China).

### Construction of microorganism bioactive materials

Firstly, 10% GelMA was prepared by mixing sterile water with 0.5% LAP (W/V). To form a uniform porous structure, a 1.6% PEO (W/V) solution was blended with the GelMA mixed solution (volume ratio of 1:1) to prepare a two-phase emulsion solution. Excited by UV light (365 nm) for 30 s, GelMA was crosslinked into hydrogel with the assistance of LAP [42]. PEO was released from hydrogel constructs by immersion in PBS buffer or yeast culture medium to produce pores in hydrogel [40]. Finally, the hydrogel construct was cultured in YPD medium at 30 °C with shaking at 160 rpm for 36 h for further investigation.

### Evaluation of cytotoxicity

The biocompatibility of different materials was assessed by live/dead staining kits. First, GelMA hydrogel and GelMA hydrogel (containing PEO) were immersed in a YPD medium for 24 h. Second, using H_2_O_2_ as a reference, *S. boulardii* was incubated with different extracted solutions derived from GelMA hydrogel and GelMA hydrogel (containing PEO). Third, *S. boulardii* was collected, cultured with PI staining solution at 37 °C for 15 min, and observed by inverted fluorescence microscopy. Furthermore, dead *S. boulardii* cells were stained in red, and B16 cells were selected as a model. Calcein-AM and PI solution were diluted to final concentrations of Calcein-AM at 2 μmol/L and PI at 4.5 μmol/L. B16 cells were collected following incubation with different components solution and dilution to a concentration of 5 × 10^5^ cells/mL. Next, B16 cells were cultured with the live/dead staining solution at 37 °C for 15 min and observed by fluorescence microscopy at excitation wavelengths of 485 and 525 nm [43]. Live cells were stained in green and dead cells were stained in red.

### Characterization of GelMA hydrogel and *S. boulardii* bioactive material

To detect the microstructure of GelMA hydrogel and the distribution of *S. boulardii* in bioactive materials, the samples were pre-cooled at −80 °C for 1 h and then freeze-dried by a freeze dryer (SCIENTZ-10N, Xinzhi, Ningbo, China) at −60 °C for 24 h. After being sprayed with conductive material by Quorum SC7620, the samples were observed using a scanning electron microscope (TescanmtraLms, Brno, Czech Republic). To improve the visualization of the pore structure, the GelMA solution was labeled with rhodamine B. After photo-crosslinking, GelMA hydrogel containing rhodamine B was soaked thoroughly. GelMA hydrogel was washed and observed using a fluorescence microscope. Likewise, the fluorescein labeling method was used to observe the activity of *S. boulardii* bioactive materials. *S. boulardii* was stained by Calcofluor White (CFW; final concentration: 10 mg/mL) for 10 min [28]. After being washed several times, *S. boulardii* bioactive materials were observed.

### Determination of intracellular ATP, MTT, and ROS; activity of antioxidative enzymes; and mitochondrial complex III / CoQ-cytochrome C reductase activity

*S. boulardii* bioactive material was decomposed by GelMA lysis buffer. First, GelMA lysis buffer (0.3 mg/mL) was added and immersed in GelMA hydrogel. The reaction took place in a 37 °C incubator with uninterrupted oscillation and was observed every 15 min. After the hydrogel was fully cleaved, the solution was centrifuged at 1000 rpm for 5 min and the supernatant was discarded. The collected *S. boulardii* was re-dispersed and quantitated by the absorbance change at 600 nm (OD_600_). The percentage encapsulation of *S. boulardii* was calculated based on the difference in OD value. The ATP content of *S. boulardii* derived from bioactive materials was determined by using ATP assay kits (Nanjing Jiancheng Bio., Nanjing, China) in strict accordance with the manufacturer’s instructions [44]. Meanwhile, an MTT assay was used to measure cell viability. The samples were mixed with MTT (0.1 mg/mL) and incubated at 37 °C for 1 h. Subsequently, the cells were collected by centrifugation and dissolved in dimethyl sulfoxide (DMSO) [45]. Absorbance was detected at 240 nm (BIO-RED, United States). Next, 2’, 7’-dichlorodihydrofluorescein diacetate (DCFH-DA) was used to measure ROS levels in cells. *S. boulardii* in bioactive materials was collected after antagonism, and the sample was adjusted to OD_600_ = 0.1. After being stained by DCFH-DA (final concentration 10 μmol/L) at 37 °C for 24 min, the sample was detected (Ex = 480 nm, Em = 520 nm) by a spectrophotometer. Intracellular superoxide dismutase (SOD1) was detected using a SOD1 assay kit (Nanjing Jiancheng Bio., Nanjing, China), and the absorbance was measured at 550 nm. According to the instructions (Nanjing Jiancheng Bio., Nanjing, China), catalase (CAT) activity and GSH content were respectively detected at 240 nm and 420 nm. The Mitochondrial complex III / CoQ-cytochrome C reductase activity was assayed at 550 nm using an assay kit (Nanjing Jiancheng Bio., Nanjing, China).

### Western blot analysis

To detect the expression level of *MCP1* gene from the perspective of protein level, western blot analysis was adapted. *S. boulardii* cultured in YPD medium was collected and washed with PBS buffer three times. Then, cells were resuspended in immunoprecipitation assay buffer (4 mol/L NaCl, 1 mol/L Tris-HCl, pH 7.5), 10% SDS, 1% NP-40, 10% C_24_H_39_O_4_Na, and 0.5 mol/L EDTA, and cell extracts were obtained. The latter was detected by sodium dodecyl sulfate polyacrylamide gel electrophoresis and transferred to polyvinylidene difluoride membranes per the standard procedures [46]. Anti-GFP (MBL598), anti-Tubulin (MBL PM054), and anti-Rabbit IgG (PROMEGA W4018) were used to enhance the luminescence intensity of the substrate. Samples were detected using a western blotting exposure meter (Tanon, 5200, multi).

### Virulence assay

Four-week-old ICR female mice (SPF) were obtained from the Jinan Pengyue Experimental Animal Breeding Co., Ltd, Jinan, China. All mice were placed in a constant environment at 24 ± 2 °C and raised in 12 h light/dark cycles wherein lights were turned on at 7:00 am. Their diet was provided under free conditions in the School of Laboratory Animal & Shandong Laboratory Animal Center. More importantly, the pain of the animals was minimized as much as possible.

Endotracheal tubes that were either blank or coated with hydrogel, bioactive materials, or bioactive materials (pGK1-*MCP1*) were incubated individually with *C. albicans* for 12 h. After being washed with PBS buffer, the tubes were implanted in the abdominal cavities of the mice. It is widely known that *C. albicans* could spread to whole organism via blood vessels, and mainly damage kidney once infecting host [47]. Therefore, three mice in each of the four groups were killed on day 7, and their kidneys were collected. Some kidneys were weighed and homogenized with PBS buffer. The diluted kidney homogenate was placed on the YPD medium. After culturing at 30 °C for 2 days, the fungal colonies were counted and standardized based on kidney weight. Four kidneys were fixed with 10% formalin for 24 h. After hematoxylin-eosin (HE) staining, the tissue sections were observed with a microscope (BX53, Olympius, Tokyo, Japan) to evaluate the degree of inflammation and tissue damage.

### Statistical analyses

The data were shown as mean ± SD from three replications. The SPSS software (version 25), ANOVA statistical analyses and Tukey’s test were applied for all bar graph figures. Statistical significance was defined based on the different *p*-values: *p < 0.05, **p < 0.01, and ***p < 0.001.

## Results

### Characterization of GelMA hydrogel

**Fig. 1:**
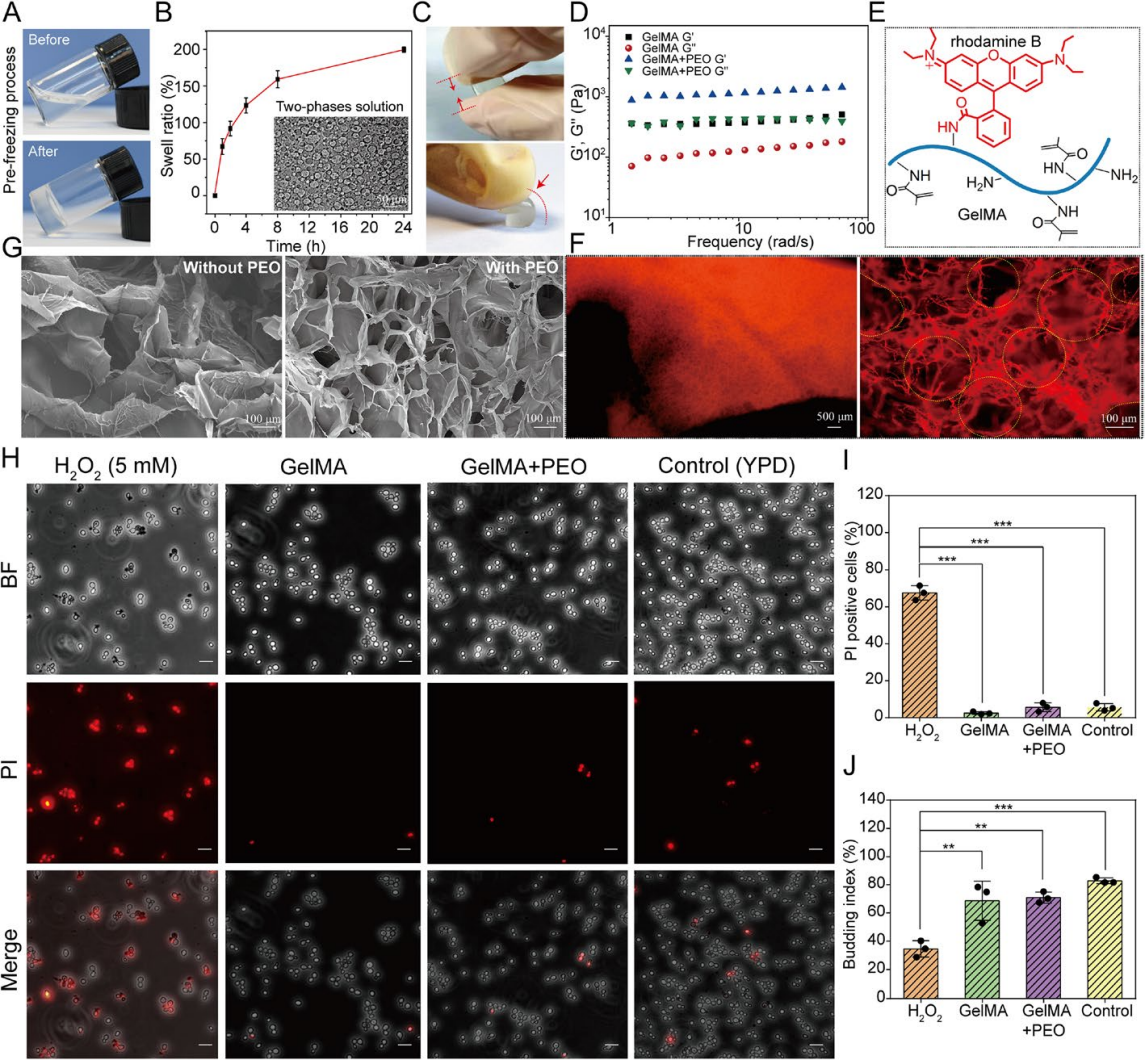
**A** States of GelMA solution before and after freezing. **B** The swelling ratio test of hydrogel immersed in water for different times after photo-crosslinking. Two-phase solution in the inset is the optical image of the emulsion morphology when the PEO concentration is 0.8%. **C** Compression and bending resistance tests of GelMA hydrogel. **D** Frequency-sweep oscillatory tests. Schematic diagram (**E**) and fluorescence images (**F**) of rhodamine B-labeled hydrogels. **G** SEM pictures of hydrogel without PEO (left) and with PEO (right). **H** Viability test of *S. boulardii*. The cells were cultured in YPD medium for 4 h. After culturing with H_2_O_2_ (5 mmol/L) and the extraction solution of GelMA and GelMA + PEO hydrogel for 24 h, the samples were stained by PI. BF (Bright field); PI (PI positive cells). Bar = 50 μm. **I** PI positive cells of *S. boulardii* cultured with different component solutions. **J** Budding rate (an indicator of cell reproductive ability) of *S. boulardii* cultured with H_2_O_2_ (5 mmol/L) and the extraction solution of GelMA and GelMA + PEO hydrogel for 24 h. *p < 0.05, **p < 0.01, and ***p < 0.001

GelMA hydrogel, integrating the characteristics of natural and synthetic biomaterials, can provide a suitable three-dimensional space for cell growth and differentiation [48]. First, the thermo-sensitive property of the GelMA solution was tested. As shown in Fig. 1A, the GelMA solution transitioned from sol state to physical gel after freezing at a low temperature (4 °C) for 30 min. The transformation process was reversible, and the physical gel state of GelMA was maintained for about 30 min at room temperature. Moreover, after mixing GelMA solution with poly(ethylene oxide) (PEO) solutions (0.8%), a boundary between two phases emerged, demonstrating microscale phase separation (inset in Fig. 1B). After UV illumination, the two-phase solution was polymerized into the hydrogel. The swelling radio of GelMA hydrogel immersed in water reached 200% at 24 h. Furthermore, the hydrogel exhibited good compressive capability and rebound performance (Figs. 1C and S2).

The rheological properties of GelMA and GelMA + PEO hydrogels were characterized by rheometer (Haake Mars60, Germany) with parallel plate (plate diameter of 20 mm) at room temperature. Frequency sweep tests shown that the elastic modulus (G’) value of GelMA and GelMA + PEO hydrogels were uniformly higher than viscous modulus (G”) without any point of crossover over the whole frequency range, meaning that both GelMA and GelMA + PEO hydrogels possessed elastic and stable gelled structure (Fig. 1D). Furthermore, the G’ and G” values of GelMA + PEO hydrogels were higher than that of GelMA hydrogel, indicating the addition of PEO enhanced the gel strength. Some reports showed that GelMA can chelate with rhodamine B based on a coupling reaction between the carboxyl groups on rhodamine B and the amino groups on GelMA [49]. Thus, rhodamine B was used as a fluorescent marker to further demonstrate the morphology of GelMA hydrogel (Fig. 1E). The results, presented in Figs. 1F and S3, showed that GelMA hydrogel contained many pores with the mean size of 113 ± 12.79 μm. SEM results, presented in Fig. 1G, further confirmed that a porous structure existed in the hydrogel in the presence of PEO (right side of the Fig. 1G) but not in the pure GelMA hydrogel (left side of the Fig. 1G).

To determine whether hydrogel damages *S. boulardii*, the biocompatibility of GelMA was detected. PI staining showed that GelMA and GelMA + PEO extracts had good compatibility with *S. boulardii*, like the control group (YPD medium as negative control groups), but unlike the H_2_O_2_ group (a cytotoxic agent as positive control groups) (Fig. 1H). Cell death rate (PI positive cells) further confirmed that *S. boulardii* maintained favorable activity in hydrogel (Fig. 1I). Moreover, the budding rate, a criterion measuring the viability of yeast, could reach 70% (Fig. 1J). Besides, B16 cells (mouse melanoma cells), as a mammalian cell model, also presented superior living status in hydrogel (Fig. S4).

### Construction and characterization of *S. boulardii* bioactive material

**Fig. 2:**
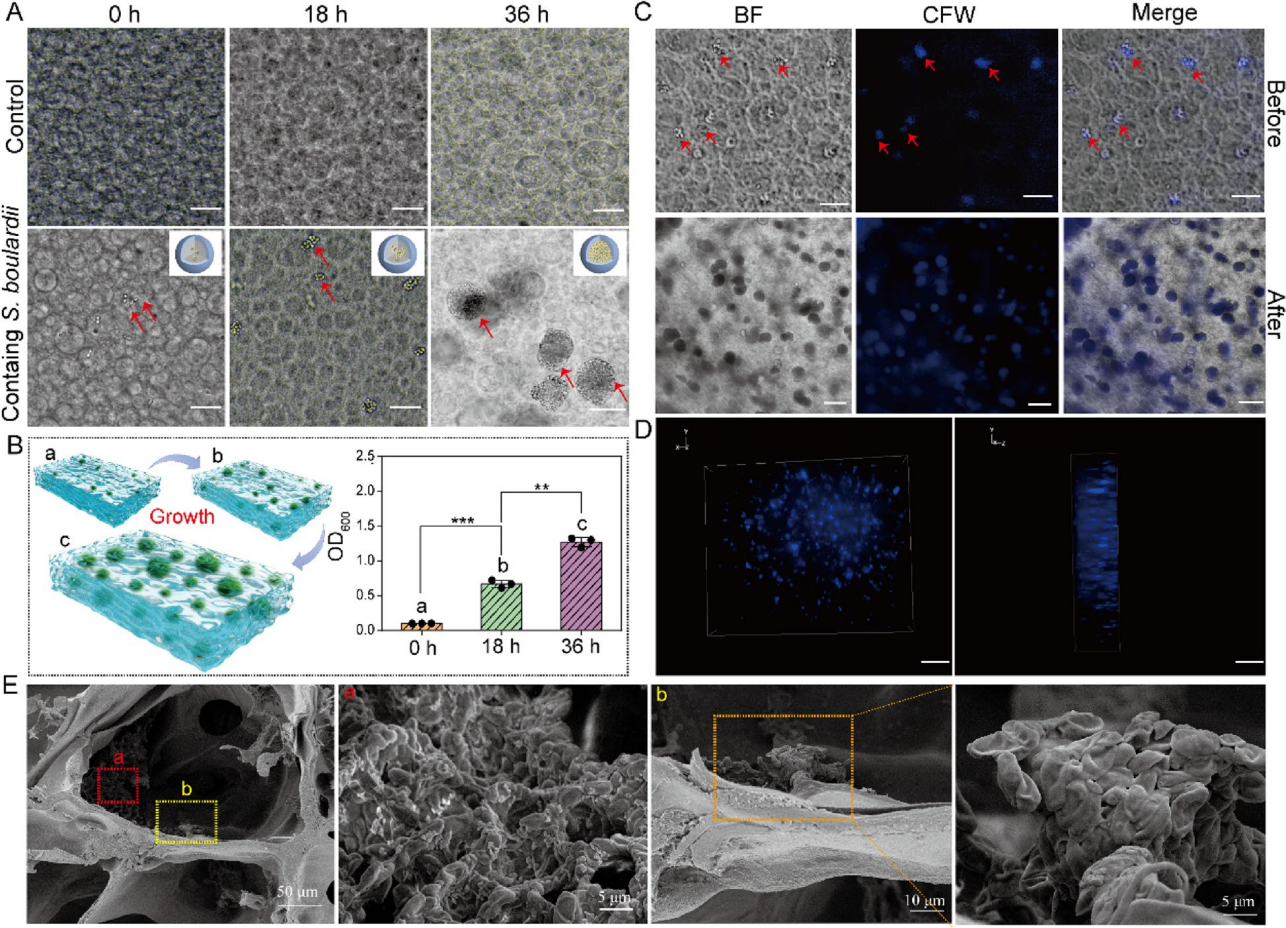
**A** Images of *S. boulardii* in bioactive material at 0 h, 18 h, and 36 h, with blank hydrogel as the control group. Bar = 50 μm. **B** Diagrammatic sketch and histogram of OD_600_ of *S. boulardii* in bioactive material. After being cultured in YPD medium for 0 h, 18 h, and 36 h, the sample was decomposed by GelMA lysis buffer for 1 h, and the released *S. boulardii* was quantitated by the absorbance change at 600 nm. **C** Fluorescence images of CFW-stained *S. boulardii* in bioactive material. *S. boulardii* in bioactive material was stained by CFW, and the distribution of *S. boulardii* at 0 h and 36 h was observed using a fluorescence microscope. Bar = 50 μm. **D** 3D reconstructed images of *S. boulardii* bioactive material obtained by multifunctional fluorescence microscope. Bar = 100 μm. **E** SEM pictures of *S. boulardii* bioactive material, where a and b represent the *S. boulardii* in hydrogel pores. *p < 0.05, **p < 0.01, and ***p < 0.001

Based on the performance of GelMA hydrogel, the *S. boulardii* bioactive material was constructed by encapsulating *S. boulardii* in the pores of GelMA via photo-crosslinking. To monitor the survival and growth of *S. boulardii* in bioactive material, images of the bioactive material at different times were recorded. Originally, *S. boulardii* was encapsulated in bioactive material at low counts, Subsequently, the amount of *S. boulardii* increased from several cells to cell clusters, and finally to large accumulations of cells that filled the hydrogel aperture (Fig. 2A). The absorbance at 600 nm (OD_600_) showed that the number of *S. boulardii* cells inside the bioactive materials increased by 12 times from 0.10 to 1.27 within 36 h (Fig. 2B). Calcofluor white (CFW) can effectively bind to the chitin of *S. boulardii* and emit blue fluorescence [5]. CFW staining showed that *S. boulardii* distributed in hydrogel pores at the initial stage. Then the number of *S. boulardii* cells confined in bioactive material increased, and an apparent cell cluster was observed (Fig. 2C and E). An all-in-one fluorescence microscopic imaging system (BZ-X800E, Keyence) further confirmed that *S. boulardii* was distributed uniformly in the bioactive material based on both the top view and side view (Fig. 2D).

**Fig. 3:**
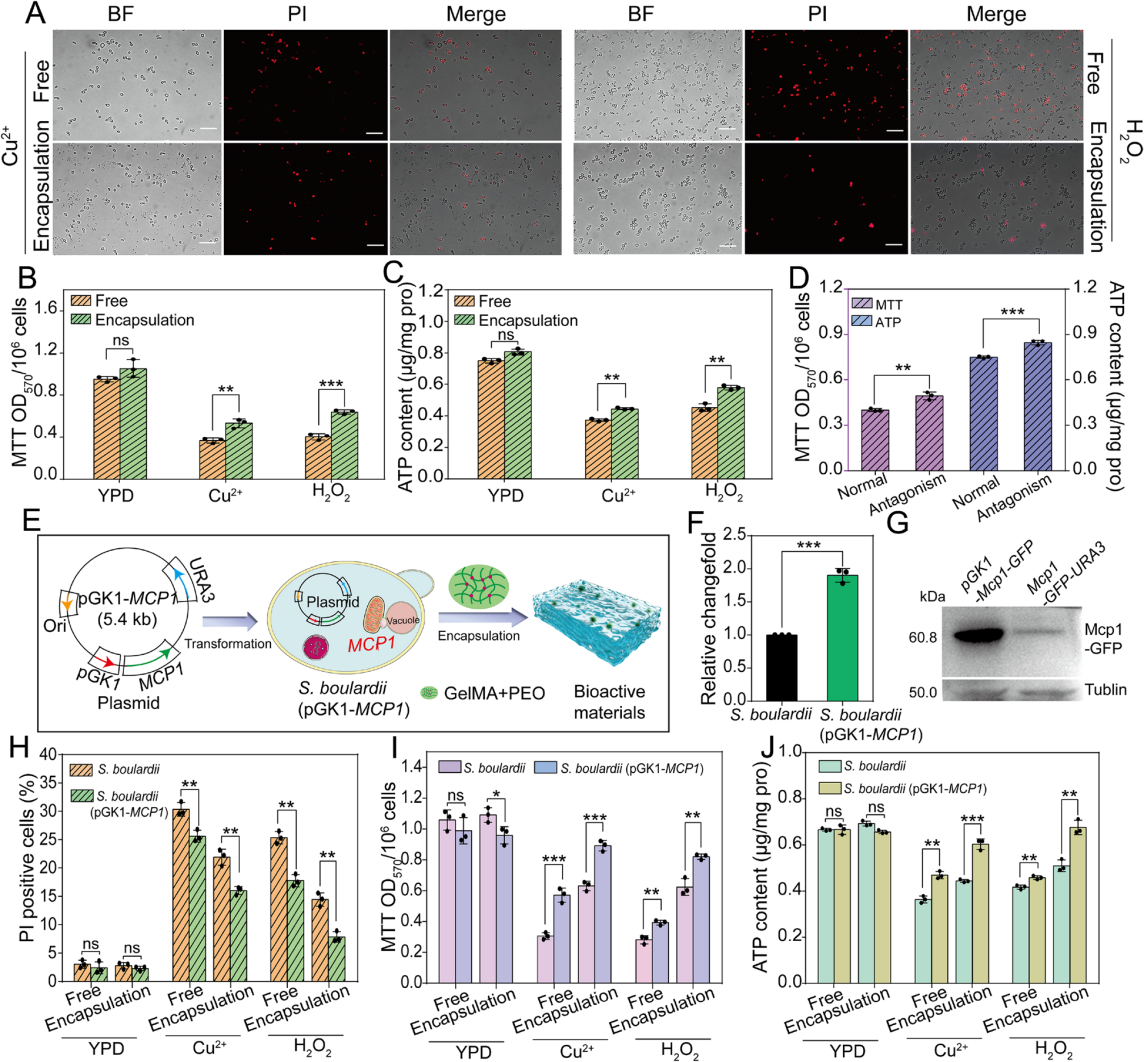
Stress resistance test of *S. boulardii* bioactive material. **A** Fluorescence images of PI-positive cells of free *S. boulardii* and *S. boulardii* bioactive material. Free *S. boulardii* and *S. boulardii* bioactive material were cultured with Cu^2+^ (2 mg/mL) and H_2_O_2_ solution (5 mmol/L) for 2 h and then stained by PI. Bar = 50 μm. Histogram analysis of **B** MTT and **C** ATP content of free *S. boulardii* and *S. boulardii* bioactive material after culturing with Cu^2+^ and H_2_O_2_ solution. **D** ATP content (right) and MTT activity (left) of *S. boulardii* bioactive material before (normal) and after (antagonism) culturing with *C. albicans* for 1 h. **E** Schematic diagram of construction process of *S. boulardii* (pGK1-*MCP1*) bioactive material. **F** Gene expression levels of *MCP1* of *S. boulardii* (pGK1-*MCP1*) analyzed by RT-PCR using *ACT1* as the normalization gene. **G** Western blotting analysis of Mcp1-GFP. After the pGK1-Mcp1-*GFP* and Mcp1-*GFP*-*URA3* strains were cultured in YPD for 4–6 h, anti-GFP was used for western blot analysis to detect Mcp1-GFP. Anti-tubulin antibody was used as the loading control to detect tubulin. **H** PI positivity rate, **I** MTT activity, and **J** ATP content of free *S. boulardii*, free *S. boulardii* (pGK1-*MCP1*), *S. boulardii* encapsulated in bioactive material, and *S. boulardii* (pGK1-*MCP1*) encapsulated in bioactive material after the samples were cultured with Cu^2+^ (2 mg/mL) and H_2_O_2_ solution (5 mmol/L) for 2 h. Statistical significance was defined based on the different *p*-values: *p < 0.05, **p < 0.01, and ***p < 0.001

To investigate the resilience of *S. boulardii* bioactive material under environmental stress, a high salt (Cu^2+^) and H_2_O_2_ solution was used as a model to observe the survival of *S. boulardii* in bioactive material. The results suggested that the mortality of *S. boulardii* in bioactive material was decreased compared to that of free *S. boulardii* (the dead cells were stained in red) (Fig. 3A).

ATP production capacity and MTT activity are important indicators of cell viability [50]. The ATP level and MTT activity of *S. boulardii* in bioactive material were higher than those of free *S. boulardii* after culturing with Cu^2+^ and H_2_O_2_ solution, especially after 2 h of incubation (Fig. 3B and C). Moreover, after culturing with *C. albicans*, *S. boulardii* in bioactive material maintained better viability according to the results of MTT and ATP assays (Fig. 3D).

### Overexpressing *MCP1* gene of *S. boulardii* bioactive material

Our previous work found that Mcp1 is involved in maintenance of mitochondrial function of eukaryotic cells under non-fermented carbon source conditions [28]. In the present study, to enhance the resistance of *S. boulardii* under environmental stress, the *S. boulardii* with overexpressed *MCP1* was constructed by transformation of the pGK1*-MCP1-URA3* plasmid and then encapsulated in hydrogel (Fig. 3E). RT-PCR results showed that the expression of *MCP1* was increased in *S. boulardii* after genetic modification (pGK1*-MCP1*) (Fig. 3F). Moreover, western blotting revealed that the transcription level of Mcp1-GFP was increased in *S. boulardii* (pGK1*-MCP1*) (Figs. 3G and S5).

To clarify the functions of overexpression of *MCP1* of *S. boulardii* under environmental stress, taking metal ions (Cu^2+^) and oxidative pressure (H_2_O_2_) as the model, the cell survival rate and mitochondrial activity of *S. boulardii* were assayed. The death rate of *S. boulardii* with overexpressed *MCP1* is lower than that of *S. boulardii*, whether in the encapsulated or free state (Fig. 3H). MTT test showed that the mitochondrial activity of *S. boulardii* was reduced, but that of *S. boulardii* (pGK1*-MCP1*) in bioactive material stayed at a high level in the presence of Cu^2+^ and H_2_O_2_ (Fig. 3I). Intriguingly, Cu^2+^ and H_2_O_2_ cannot affect the ATP production of *S. boulardii* with overexpressed *MCP1* in bioactive material (Fig. 3J). The bioactive materials as endotracheal tube coating will be placed in endotracheal lumen, thus, a leaching test of *S. boulardii* encapsulated in coating was carried out to assess the potential impact on lung. To mimic physiological environment in vivo, the bio-coatings containing *S. boulardii* and transgenic *S. bourlardii* were immersed in physiological saline for 4 days, respectively. Then, the soaking solution was spread on agar broth plates to culture for 24 h. As shown in Fig. S6, a large amount of *S. bourlardii* were observed in GelMA groups, indicating *S. boulardii* leaked from the GelMA hydrogel. However, few *S. bourlardii* growth were found in GelMA + PEO groups, suggesting hardly no *S. boulardii* can escape from hydrogel under physiological saline condition. In addition, the survival rate of encapsulated *S. boulardii* was assayed when the disk-shaped hydrogels encapsulating *S. boulardii* and transgenic S. *boulardii* were placed in abdominal cavity of mice (Fig. S7A). After 4 days, the disk-shaped hydrogels were taken out and lysed by GelMA lysis buffer. The obtained *S. boulardii* and transgenic *S. bourlardii* were stained with Calcein-AM/PI. Both *S. boulardii* and transgenic *S. boulardii* encapsulated in hydrogel have a good growth status (Fig. S7B). The cell survival rate of them was more than 94% after implanted in vivo for 4 days. These results indicated that overexpressing the *MCP1* gene of *S. boulardii* and encapsulating it in bioactive materials can enhance the resistance of *S. boulardii* to environmental stress.

### Anti-adhesion of endotracheal tube coating against *C. albicans*

**Fig. 4:**
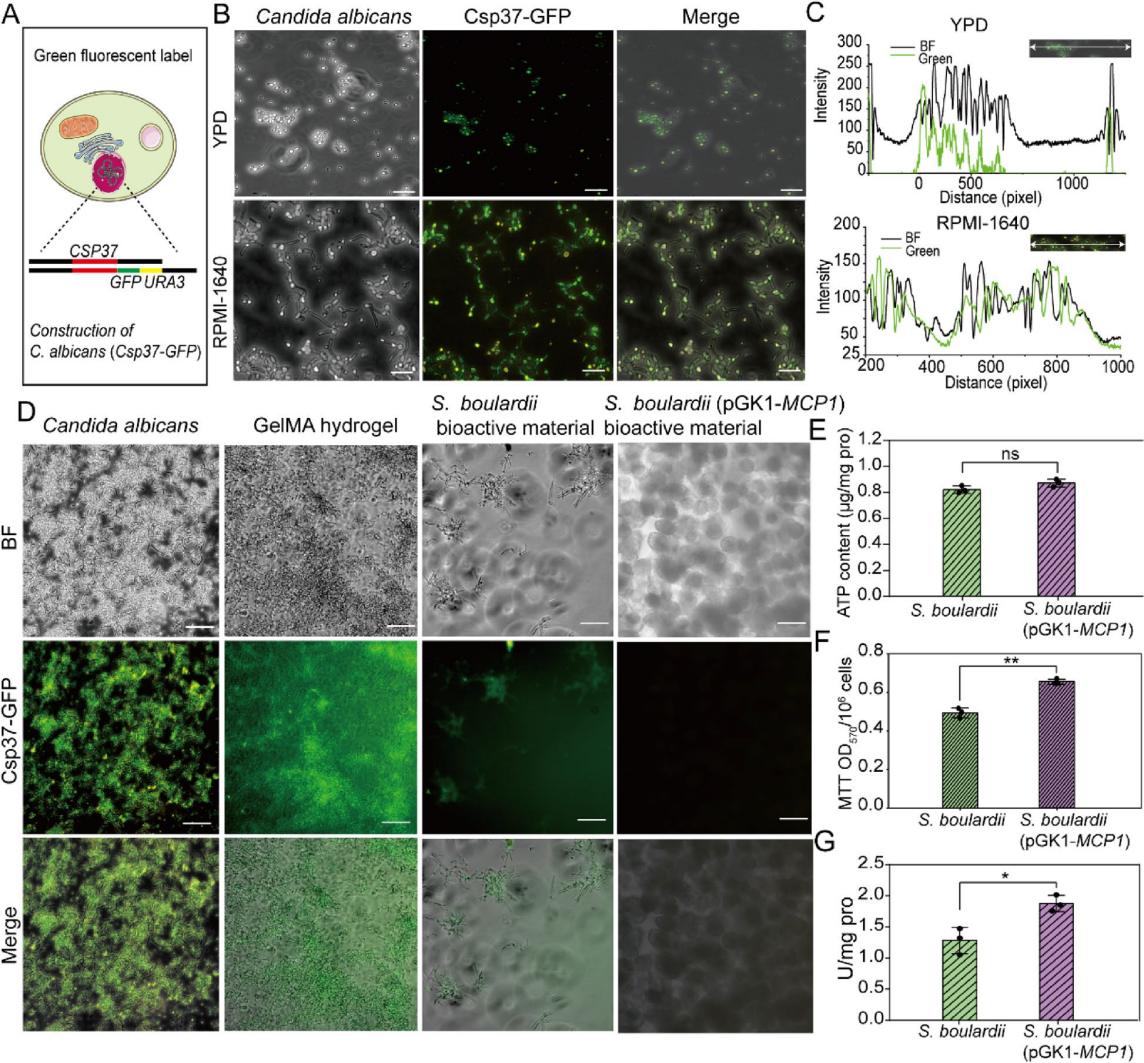
**A** Schematic diagram of GFP-labeled *C. albicans*. **B** Fluorescence images of GFP-labeled *C. albicans*. The *C. albicans* (Csp37-GFP) was cultured in YPD medium and RPMI-1640 medium for 4 h and then observed by fluorescence microscope. Bar = 50 μm. **C** ImageJ analysis of GFP-labeled *C. albicans* after being cultured in YPD medium and RPMI-1640 medium. **D** Antagonism of tube coating (*S. boulardii* bioactive material) against *C. albicans* adhesion. The tube coatings of GelMA hydrogel, *S. boulardii* bioactive material, and *S. boulardii* (pGK1-Mcp1) bioactive material were cultured with *C. albicans* (Csp37-GFP) for 2 h and then detected by fluorescence microscope. Bar = 50 μm. **E** ATP contents and **F** MTT test results of *S. boulardii* and *S. boulardii* (pGK1-Mcp1) in bioactive material after culturing with *C. albicans* for 2 h. **G** Mitochondrial respiratory chain complex III of *S. boulardii* and *S. boulardii* (pGK1-*MCP1*) in bioactive material after culturing with *C. albicans* for 2 h. Statistical significance was defined based on the different *p*-values: *p < 0.05, **p < 0.01, and ***p < 0.001

*C. albicans* can easily adhere to the surface of an endotracheal tube, potentially causing fungal infections, especially in severe patients or people with compromised immune systems [10]. In the present study, *S. boulardii* bioactive materials were chosen to coat the endotracheal tube to inhibit the adhesion of *C. albicans*. To distinguish *S. boulardii* and *C. albicans* (after antagonism), GFP-labeled *C. albicans* (WT-*CSP37-GFP*) was constructed by lithium acetate transformation (Fig. 4A). In the experiment, green fluorescence could be observed in *C. albicans* both in yeast and mycelium morphology (Fig. 4B). Analysis results of ImageJ further confirmed that *C. albicans* (WT-*CSP37*-*GFP*) was successfully constructed (Fig. 4C).

The *S. boulardii* bioactive materials were used to coat an endotracheal tube and then the antagonistic activity of the materials towards *C. albicans* was assessed. The adhesion of *C. albicans* was significantly reduced when the tube surface was covered with *S. boulardii* (pGK1*-MCP1*) bioactive material (Fig. 4D). Furthermore, the ATP level and MTT activity of *S. boulardii* (pGK1*-MCP1*) in hydrogel remained high after antagonism (Fig. 4E and F). Strikingly, the activity of mitochondrial respiratory chain complex III of *S. boulardii* (pGK1*-MCP1*) increased significantly after antagonism, which confirmed that the activity of mitochondria was enhanced (Fig. 4G). It is worth noting that sterilization processes are essential for medical devices. In clinical practice, most endotracheal tubes are made of polyvinyl chloride (PVC) [2, 51]. Ethylene oxide gas is commonly used for sterilization due to its effectiveness and compatibility with PVC [52, 53]. In this study, sterilized endotracheal tubes were coated with *S. boulardii* bioactive material in a sterile environment and exposed to UV radiation for further sterilization treatment [54]. Additionally, the survival of *S. boulardii* under UV irradiation was investigated. As shown in Fig. S8, both *S. boulardii* and *S. boulardii* bioactive material used as a tube coating maintained good activity under different irradiation times.

**Fig. 5:**
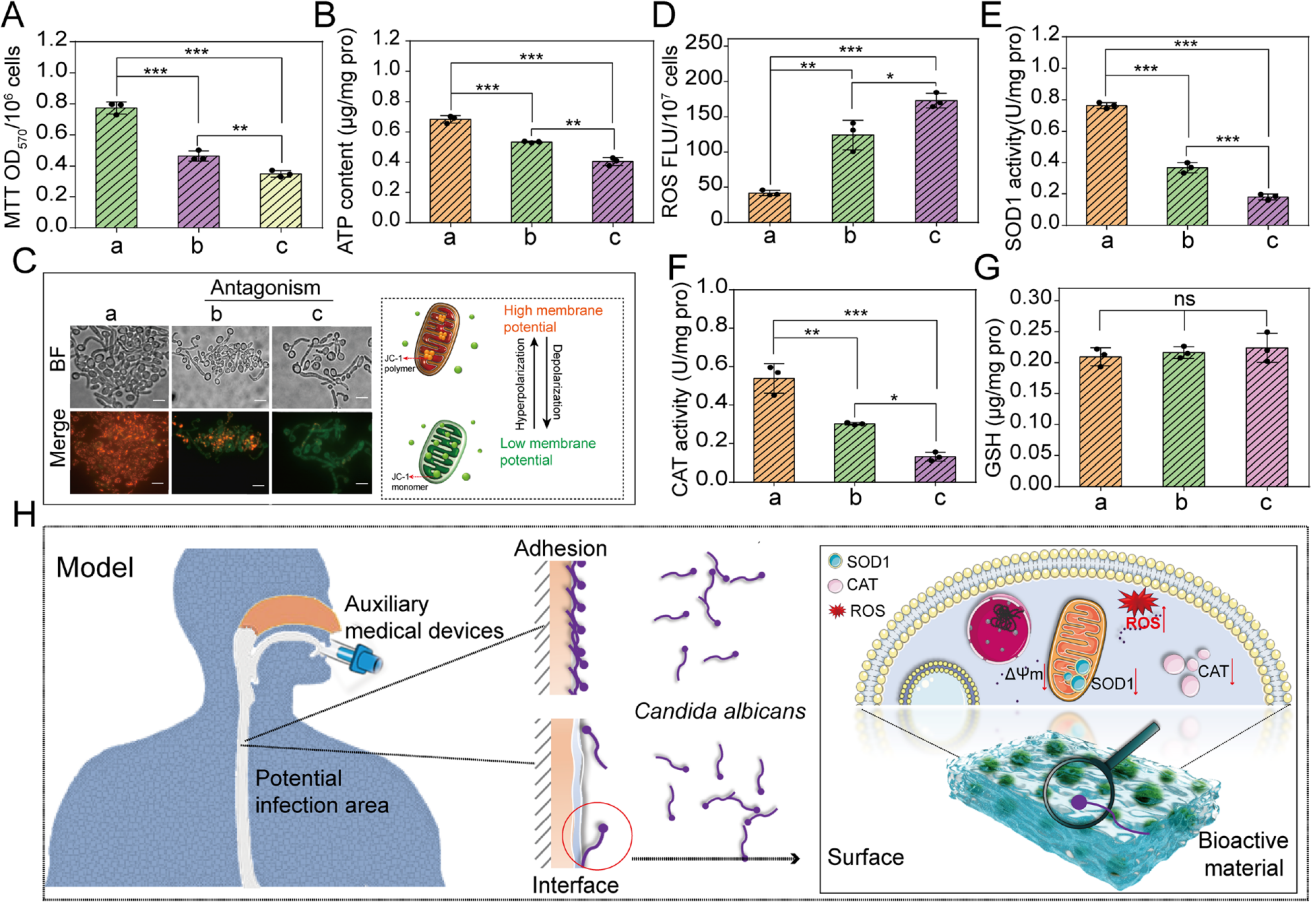
**A** MTT activity and **B** ATP content of *C. albicans* (a) before being cultured, (b) after being cultured with *S. boulardii* bioactive material, and (c) after being cultured with *S. boulardii* (pGK1-Mcp1) bioactive material for 2 h. **C** Fluorescent images and schematic diagram of mitochondrial membrane potential (Ψm) of *C. albicans* before and after being cultured with *S. boulardii* bioactive material and then stained by JC-1. (a), (b), and (c) are the same as in (**A**). Bar = 50 μm. **D** ROS, **E** SOD1, **F** CAT, and **G** GSH of *C. albicans* before and after antagonism. (a) Control, (b) culturing with *S. boulardii* bioactive material, and (c) culturing with *S. boulardii* (pGK1-*MCP1*) bioactive material. **H** Schematic diagram of antagonistic mechanism of *S. boulardii* (pGK1-*MCP1*) bioactive material against *C. albicans*. Statistical significance was defined based on the different *p*-values: *p < 0.05, **p < 0.01, and ***p < 0.001

To explore the impact of *S. boulardii* (pGK1-*MCP1*) bioactive material on *C. albicans*, the MTT activity and ATP content of *C. albicans* were assayed. It was found that MTT activity and ATP levels both decreased obviously (Fig. 5A and B). Additionally, some previous research demonstrated that the change of mitochondrial membrane potential (Ψm) can reflect mitochondrial function [55, 56]. JC-1 as fluorescent probe was used to detect Ψm of *C. albicans*. When Ψm of mitochondria is high, JC-1 aggregates in the matrix of mitochondria to form polymers (JC-1 polymer), resulting in the production of red fluorescence. When Ψm is low, JC-1 cannot be gathered in the mitochondrial matrix, resulting in the production of green fluorescence. Herein, JC-1 staining showed that *C. albicans* emitted obvious green fluorescence after being antagonized, indicating that the Ψm of mitochondria was low (Fig. 5C). These results indicated that the mitochondrial functions of *C. albicans* were disorderly after being cultured with the tube coating (*S. boulardii* bioactive material).

It is widely known that the functional stability of mitochondria plays an important role in the maintenance of intracellular ROS levels, which influences the oxidative response. To investigate whether mitochondria were damaged, intracellular ROS and the activity of antioxidant enzymes in *C. albicans* after antagonism were investigated. The increased ROS, the lowered activity of intracellular SOD1 and CAT, and relatively stable GSH content of *C. albicans* after antagonism (Fig. 5D, E, F, and G) revealed that the oxidative response system of *C. albicans* was destroyed. These results proved that *S. boulardii* (pGK1-*MCP1*) bioactive material can be used as a coating for an endotracheal tube to prevent *C. albicans* adhesion by damaging the oxidation response system of *C. albicans* (Fig. 5H).

### Investigation of tissue invasion of *C. albicans*

**Fig. 6:**
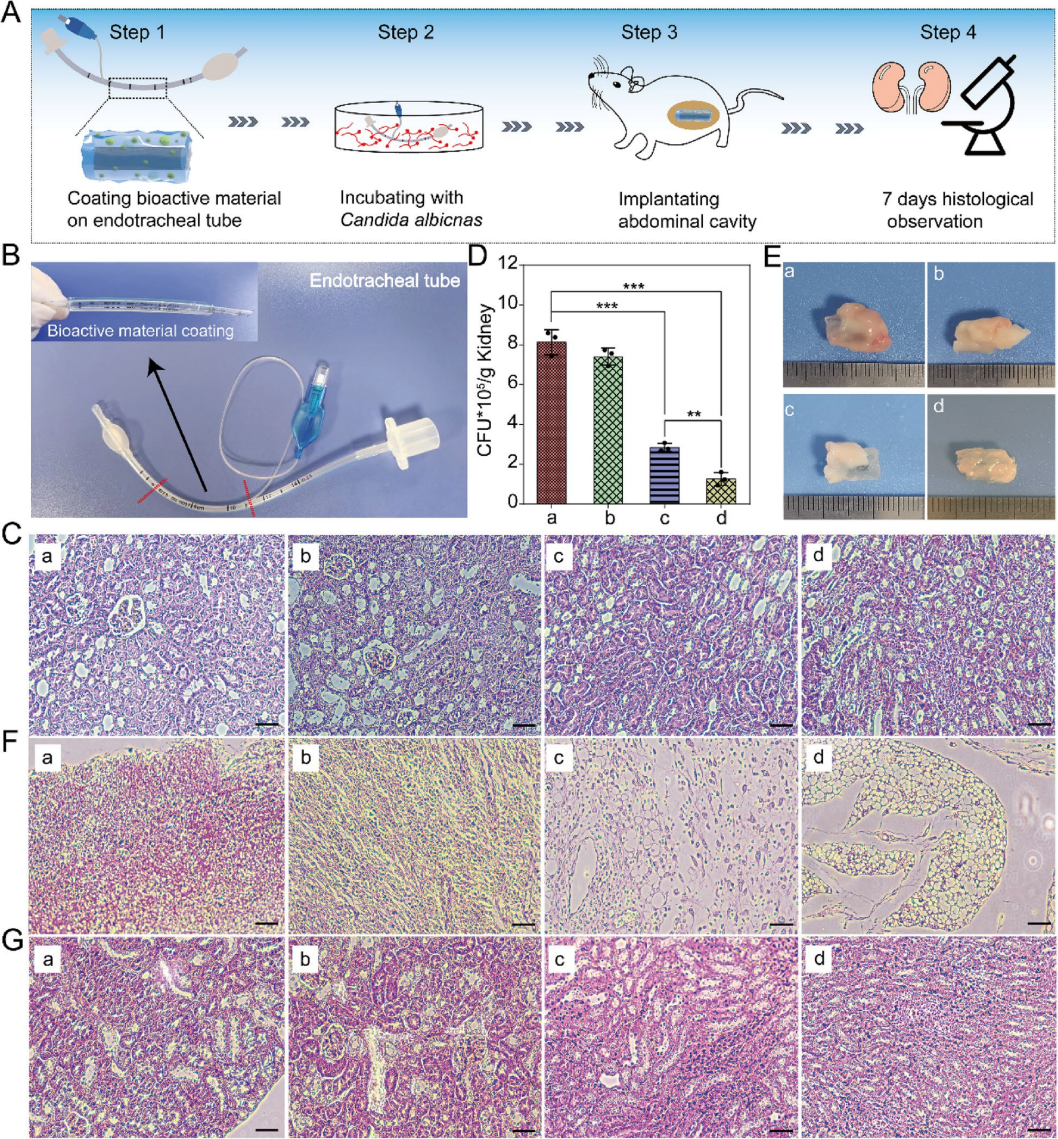
**A** Schematic diagram of *S. boulardii* bioactive material used as coating of endotracheal tube to inhibit the ability of *C. albicans* to infect mice. **B** Photos of the uncoated endotracheal tube and endotracheal tube coated with *S. boulardii* bioactive material. Endotracheal tube was immersed in GelMA solution containing PEO, LAP, and *S. boulardii*, and then illuminated by UV at Ex = 365 nm to finish photo-crosslinking. **C** Tissue sections of mice kidneys surgically implanted with tubes that were (a) blank or (b) coated with hydrogel, (c) coated with *S. boulardii* bioactive material, or (d) coated with *S. boulardii* (pGK1-*MCP1*) bioactive material for 7 days. **D** The capacity of *C. albicans* in kidney. (a), (b), (c), and (d) are the same as in (**C**). **E** Photos and **F** attachment tissue section of tube and tubes coated with different materials derived from mice. **G** Tissue sections of kidneys of mice after implantation of uncoated tube or tubes coated with different materials cultured with *C. albicans* for 2 h. (a) Control tube, (b) tube coated with hydrogel, (c) tube coated with *S. boulardii* bioactive material, and (d) tube coated with *S. boulardii* (pGK1-*MCP1*) bioactive material. Bar = 100 μm. Statistical significance was defined based on the different *p*-values: *p < 0.05, **p < 0.01, and ***p < 0.001

Once it infects a host organism, *C. albicans* can spread throughout the organism via blood vessels and cause kidney damage [47]. To further study the ability of an *S. boulardii* bioactive material tube coating to antagonize *C. albicans*, a tissue invasion experiment was executed (Fig. 6A). Through immersion and photo-crosslinking, tubes coated with hydrogel, bioactive material, and bioactive material (pGK1-*MCP1*) were constructed (Fig. 6B). These tubes were surgically implanted into the abdominal cavities of mice. Analysis of the tissue sections of the kidneys revealed that none of the endotracheal tubes caused inflammation, indicating that both hydrogel and bioactive material offered acceptable biocompatibility (Fig. 6C).

After culturing with *C. albicans*, the uncoated endotracheal tube and endotracheal tube coated with hydrogel induced clear inflammation (Fig. 6E), and the content of *C. albicans* in the kidney was high (Fig. 6D). However, the tubes coated with *S. boulardii* bioactive material and *S. boulardii* (pGK1-*MCP1*) bioactive material induced much less inflammation, and the content of *C. albicans* in the kidney was obviously reduced (Fig. 6D, F and G).

## Discussion

The retention of endotracheal tube may result in the adhesion and biofilm formation of *C. albicans*, inducing *Candida* infection. *S. boulardii*, can inhibit the adhesion and hyphae development of *C. albicans* [25], is a promising alternative therapy for preventing *C. albicans* infection. However, *S. boulardii* cannot colonize the surface of an endotracheal tube, and it is easily affected by external conditions. In this work, PEO as a porogen endowed GelMA hydrogel with a porous structure based on two-phase emulsion, and *S. boulardii* could grow, proliferate, and form aggregates in the hydrogel pores. Therefore, GelMA + PEO can be used as carriers for microorganisms. *S. boulardii*, which proliferates by budding, is a typical case of the planktonic growth type. This raises the following question: why does *S. boulardii* aggregate in bioactive materials?

First, the cavity space is larger than that in a hydrogel, promoting the accumulation of metabolized nutrients. Meanwhile, the pore is like a “house” that protects *S. boulardii* from external interference. Furthermore, growth in confined spaces can prevent daughter cells from escaping and increases the chance of cell collisions, resulting in the formation of cell aggregates [57]. Some researchers also pointed out that confined spaces could promote the transition of a microorganism from a fluid-like state to a solid-like state [58]. In addition, in view of the thermal-sensitive property of GelMA and the lower swelling rate of GelMA hydrogel, GelMA could be used as bioink in 3D bioprinting to construct customized coatings for medical devices.

Interestingly, the encapsulation method enhanced the ability of *S. boulardii* to resist environmental stress, most likely because the hydrogel acted as a “house” protecting *S. boulardii* from hostile conditions. Moreover, quorum sensing derived from aggregated microorganisms can strengthen the resilience of probiotics. Further study revealed that *S. boulardii* bioactive material can inhibit the adhesion of *C. albicans* by damaging its mitochondria. However, the antagonistic ability of *S. boulardii* bioactive material needs further improvement.

Mitochondrial function is crucial for maintaining cell viability [59]. The mitochondrial membrane protein Mcp1 helps maintain mitochondrial function by recruiting Vps13 [26]. Our previous research also found that Mcp1 plays an important role in protecting mitochondrial functions from external pressure. Therefore, *S. boulardii* (pGK1-*MCP1*) was constructed and encapsulated in hydrogel in this work. When cultured with metal ions and H_2_O_2_, the cell death rate of *S. boulardii* (pGK1-*MCP1*) decreased obviously compared with that of *S. boulardii*. Strikingly, *S. boulardii* (pGK1-*MCP1*) bioactive material as the coating of an endotracheal tube efficiently inhibited the adhesion of *C. albicans*, more so than that of the *S. boulardii* bioactive material. In the mice model of systemic infection, *S. boulardii* (pGK1-*MCP1*) bioactive material did not cause inflammation and reduced the fungal burden of the kidney, meaning that the bioactive material coating can reduce the probability of *C. albicans* infections. Therefore, *S. boulardii* bioactive materials can feasibly be used as a coating in clinical application.

To investigate the specific role of *MCP1* in conferring resistance to environmental stress, mitochondrial functions of *S. boulardii* (pGK1-*MCP1*) were detected. Overexpression of *MCP1* enhanced the ATP level and MTT activity of *S. boulardii*. Additionally, RT-PCR results indicated that the gene expression levels of the F1F0 ATP synthase gene (*ATP15*) and succinate dehydrogenase 4 (*SHH4*) were increased. These results demonstrated that *MCP1* could strengthen mitochondrial functions, which was consistent with the findings of a previous study [27]. Apart from ATP production, mitochondrial function is closely related to the activity of the respiratory chain. As expected, overexpression of *MCP1* improved respiratory chain activity. Furthermore, the expression levels of downstream genes of the electron transport chain-including cytochrome c subtype 1 (*CYC1*), cytochrome c subtype 2 (*CYC2*), cytochrome c1 (*CYT1*), and inner mitochondrial membrane protein 3 (*SDH3*) (Fig. S9)-were detected. The rising expression level confirmed that overexpression of *MCP1* promoted electron transfer of the mitochondrial electron transport chain. Accordingly, we infer that *MCP1* enhanced respiratory chain activity by accelerating electron transfer, and it further amplified mitochondrial function, so *S. boulardii* (pGK1-*MCP1*) exhibited favorable stress-tolerant. Currently, many strategies have been explored to reduce or prevent the attachment of fungal. One is based on releasing antifungal drugs from medical devices, but it has short-lived efficacy. The other is based on permanently binding drug onto the surfaces of medical devices. The antifungal activity may be affected due to the binding drug cannot freely diffusible into the cytosol of fungal cells. Conversely, since probiotics maintain good cellular activity in the hydrogel for a long time, bioactive coating can achieve the purpose of safe, effective, and perdurable antibacterial.

Furthermore, the following question must also be explored: why can *S. boulardii* bioactive material inhibit the adhesion of *C. albicans*? Relevant literature reports that the cell activity of *C. albicans* is closely related to mitochondrial functions. In this work, mitochondrial functions of *C. albicans* were tested by measuring the membrane potential, ATP level, and MTT activity. The results showed that the mitochondrial functions of *C. albicans* were significantly damaged. In addition, mitochondria, as the main sites of ROS production and clearance, are susceptible to ROS level, which depends on the activity of antioxidant enzymes [60–62]. The high ROS level and decreased activities of SOD1 and CAT suggested that the *S. boulardii* (pGK1-*MCP1*) bioactive material coating impaired the mitochondrial functions of *C. albicans*. This may be because the overexpression of *MCP1* enhances the mitochondrial function of *S. boulardii*, promotes the secretion of secondary metabolites, and impairs the activity of antioxidant enzymes in *C. albicans*.

## Conclusion

In this study, *S. boulardii* bioactive material was successfully constructed. It was found that the antagonistic capacity and resilience of *S. boulardii* were enhanced by overexpressing the *MCP1* gene and employing hydrogel as a “house” to encapsulate *S. boulardii* through a one-step photo-crosslinked method. When used to coat an endotracheal tube, *S. boulardii* bioactive material could damage the mitochondrial functions and activity of antioxidant enzymes (SOD1 and CAT) of *C. albicans*. Furthermore, *S. boulardii* in bioactive material retained good viability after antagonizing *C. albicans*. Our proposed dual enhancement mode of *S. boulardii*, based on the confined growth of *S. boulardii* and the overexpression of *MCP1*, could broaden the application of yeasts as medical device coatings to prevent fungal infections.

### Supplementary Information

**Additional file 1: Figure S1.** (A) *S. boulardii *inhibit adhesion of *C. albicans. S. boulardii *and *C. albicans *were mixed and cultured in polyethylene culture dish for 1 h. Bar = 10 μm. (B) Crystal violet staining method was used to determine the adhesion of *C. albicans*. (C) *S. boulardii *inhibition the hyphal development of *C. albicans*. Bar = 10 μm. (D) Determination the growth of hyphal. The length of the hypha is measured by Image J. Statistical significance was defined based on the different *p*-values: *p < 0.05, **p < 0.01, and ***p < 0.001. **Figure S2.** Compression and bending resistance tests of GelMA hydrogel. **Figure S3.** (A) The images of GelMA + PEO hydrogel and rhodamine B-labelled GelMA + PEO hydrogel. (B) Pore size distribution of GelMA+PEO hydrogel. **Figure S4.** Viability determination of B16 cells. The Calcein-AM, PI staining and merge images of B16 cells cultured respectively with DMSO and the extraction solution of GelMA and GelMA + PEO hydrogel. (Green remarked live cells, red remarked dead cells). Bar = 50 μm. **Figure S5.** (A) A full scan of Fig. 3G (Mcp1-GFP) entire original gel; (B) A full scan of Fig. 3G (Tubulin) entire original gel. **Figure S6.** Leaching test of *S. boulardii* encapsulated in coating (A) The images of bio-coatings containing *S. boulardii* and transgenic *S. bourlardii* were immersed in physiological saline (0.9% NaCl) for 4 days, respectively. (B) Photos of *S. boulardii* and transgenic *S. bourlardii* colonies on agar broth plates separated from the soaking solution with different bio-coating. **Figure S7.** In vivo implanted studies (A) Photos of *S. boulardii *hydrogels in vitro and in vivo, and the attachment tissue sections with different hydrogels derived from mice. (B) The cell survival rate of *S. boulardii* and transgenic S.* boulardii *encapsulated in hydrogels after implanted in vivo for 4 days. Statistical significance was defined based on the different *p*-values: *p < 0.05, **p < 0.01, and ***p < 0.001.** Figure S8.** (A) Calcein-AM/PI staining of *S. boulardii* in bioactive materials under condition of UV at 180 min. (Green remarked live cells, red remarked dead cells) Bar = 50 μm. (B) The cells survival rate of *S. boulardii* in bioactive materials under condition of UV at 30, 60, 120, and 180 min. Statistical significance was defined based on the different *p*-values: *p < 0.05, **p < 0.01, and ***p < 0.001.** Figure S9.** Overexpression of *MCP1* enhances mitochondrial function of *S. boulardii* in bioactive materials after Co-incubation with *C. albicans*. (A) The expression levels of mitochondrial related genes *ATP15* and *SHH4* of *S. boulardi *revealed by RT-PCR. Expression levels of electron transport chain related genes *CYC1*, *CYC7*, *CYT1* and *SDH3* revealed by RT-PCR. *ACT1 *was used as the normalization gene. Statistical significance was defined based on the different *p*-values: *p < 0.05, **p < 0.01, and ***p < 0.001.

## Data Availability

The data and analysis generated in this study are included in the manuscript and Supporting Information file.

## Abbreviations

*C. albicans*: *Candida albicans*
*S. boulardii*: *Saccharomyces boulardii*
Mcp1: Mitochondrial outer membrane protein10 complementing protein1
GelMA: Gelatin methacrylates
PEO: Polyethylene oxide
PI: Propidium iodide
MTT: 3-(4,5-dimethylthiazol-2-yl)-2,5- diphenyltetrazolium bromide
GFP: Green fluorescent protein
ATP: Adenosine-triphosphate
LAP: Lithium phenyl (2, 4, 6-trimethylbenzoyl) phosphinate
B16 cells: Mouse melanoma cells
YPD: Yeast extract peptone dextrose
PBS: Phosphate buffered saline
PCR: Polymerase chain reaction
SDS-PAGE: Sodium dodecyl sulfate polyacrylamide gel electrophoresis
Csp37: Mitochondrial outer membrane protein
Tris-HCl: Tris(hydroxymethyl)aminomethane hydrochloride
CFW: Calcofluor white
NP-40: Octylphenoxypolyethoxyethanol
SDS: Sodium dodecyl sulfate
EDTA: Ethylene diamine tetraacetic acid
SEM: Scanning electron microscopes
OD600: Absorbance change at 600 nm
Ψm: Mitochondrial membrane potential
ROS: Reactive oxygen species
SOD1: Superoxide dismutase
CAT: Catalase
GSH: Glutathione
Cu^2+^: Copper ion
H_2_O_2_: Hydrogen peroxide
