# Supplementary material for: Antifungal bio-coating of endotracheal tube built by overexpressing the MCP1 gene of Saccharomyces boulardii and employing hydrogel as a “house” to antagonize Candida albicans
Source: Biomater Res. 2023 Oct 5;27:97. doi: 10.1186/s40824-023-00443-1 (PMC10557164; doi:10.1186/s40824-023-00443-1)
Supplement: Supplementary file 1 — Additional file 1: Figure S1. (A) S. boulardii inhibit adhesion of C. albicans. S. boulardii and C. albicans were mixed and cultured in polyethylene culture dish for 1 h. Bar = 10 μm. (B) Crystal violet staining method was used to determine the adhesion of C. albicans. (C) S. boulardii inhibition the hyphal development of C. albicans. Bar = 10 μm. (D) Determination the growth of hyphal. The length of the hypha is measured by Image J. Statistical significance was defined based on the different p-values: *p < 0.05, **p < 0.01, and ***p < 0.001. Figure S2. Compression and bending resistance tests of GelMA hydrogel. Figure S3. (A) The images of GelMA + PEO hydrogel and rhodamine B-labelled GelMA + PEO hydrogel. (B) Pore size distribution of GelMA+PEO hydrogel. Figure S4. Viability determination of B16 cells. The Calcein-AM, PI staining and merge images of B16 cells cultured respectively with DMSO and the extraction solution of GelMA and GelMA + PEO hydrogel. (Green remarked live cells, red remarked dead cells). Bar = 50 μm. Figure S5. (A) A full scan of Fig. 3G (Mcp1-GFP) entire original gel; (B) A full scan of Fig. 3G (Tubulin) entire original gel. Figure S6. Leaching test of S. boulardii encapsulated in coating (A) The images of bio-coatings containing S. boulardii and transgenic S. bourlardii were immersed in physiological saline (0.9% NaCl) for 4 days, respectively. (B) Photos of S. boulardii and transgenic S. bourlardii colonies on agar broth plates separated from the soaking solution with different bio-coating. Figure S7. In vivo implanted studies (A) Photos of S. boulardii hydrogels in vitro and in vivo, and the attachment tissue sections with different hydrogels derived from mice. (B) The cell survival rate of S. boulardii and transgenic S. boulardii encapsulated in hydrogels after implanted in vivo for 4 days. Statistical significance was defined based on the different p-values: *p < 0.05, **p < 0.01, and ***p < 0.001. Figure S8. (A) Calcein-AM/PI staining of S [file 40824_2023_443_MOESM1_ESM.docx]

**Support information**

Antifungal bio-coating of endotracheal tube built by overexpressing the MCP1 gene of Saccharomyces boulardii and employing hydrogel as a “house” to antagonize Candida albicans

Authors and affiliations

Yunyun Wei^1,3^, Jianfeng Qiu^3, 5^, Ziqiang Han^2^, Xuanyi Wang^4^, Hui Zhang^2^, Xinya Hou^4^, Xiangwei Lv^4^, Xiaolong Mao^2*^

^1^ School of Radiology, The Second Affiliated Hospital of Shandong First Medical University, Tai'an, China.

^2^ School of Laboratory Animal & Shandong Laboratory Animal Center, Shandong First Medical University & Shandong Academy of Medical Sciences, Shandong, Jinan, China.

^3^ School of Radiology, Shandong First Medical University and Shandong Academy of Medical Sciences, Tai'an, China.

^4^ Department of Clinical Medicine, Shandong First Medical University and Shandong Academy of Medical Sciences, Jinan, China.

^5^ Science and Technology Innovation Center, Shandong First Medical University & Shandong Academy of Medical Sciences, Jinan, China.

^*^Corresponding authors

Xiaolong Mao

E-mail: fudan671@163.com

**Material**

Gelatin methacrylates (GelMA), lithium phenyl (2, 4, 6-trimethylbenzoyl) phosphinate and GelMA lysis buffer (Suzhou Yongqinquan Intelligent Equipment Co., Ltd, Suzhou, China). Mouse melanoma cells (B16 cells) (Shanghai Qida Biotechnology Co., Ltd, Shanghai, China). Poly(ethylene oxide) (Shanghai Macklin Biochemical Co., Ltd ,Shanghai, China). Calcein-AM/propidium and Rhodamine B (Beijing Solarbio Science & Technology Co., Ltd, Beijing, China). Copper standard solution (Beijing Haibin Hongmeng Standard Material Technology Co. LTD, Beijing, China).

**
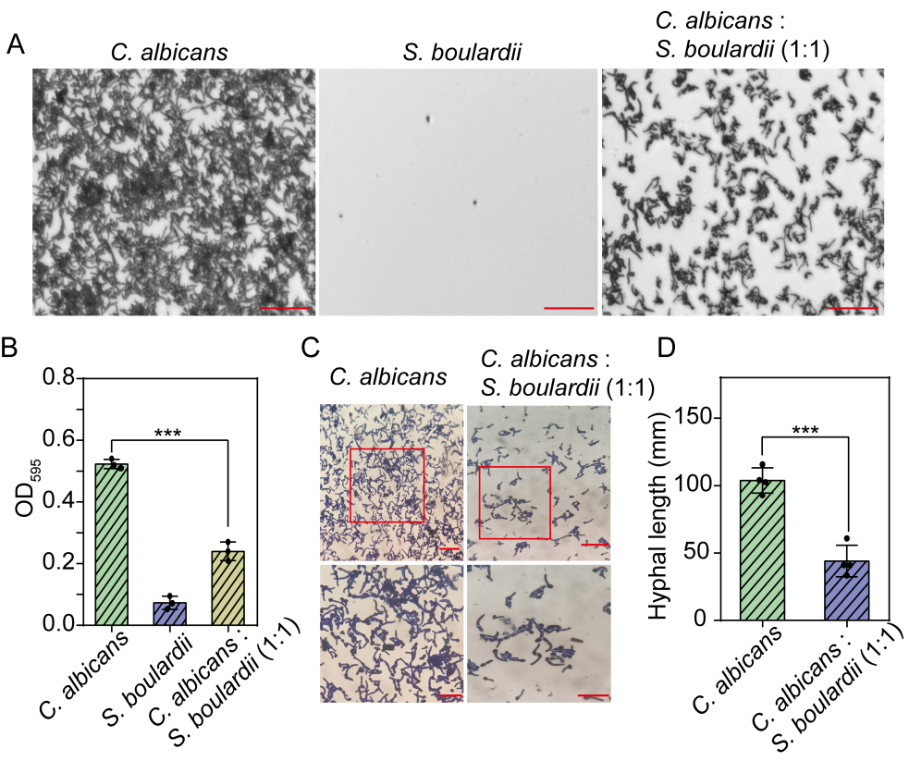
**

**Figure S1.** (A) *S. boulardii* inhibit adhesion of *C. albicans. S. boulardii* and *C. albicans* were mixed and cultured in polyethylene culture dish for 1 h. Bar = 10 μm. (B) Crystal violet staining method was used to determine the adhesion of *C. albicans*. (C) *S. boulardii* inhibition the hyphal development of *C. albicans*. Bar = 10 μm. (D) Determination the growth of hyphal. The length of the hypha is measured by Image J. Statistical significance was defined based on the different *p*-values: **p*<0.05, ***p*<0.01, and ****p*<0.001.


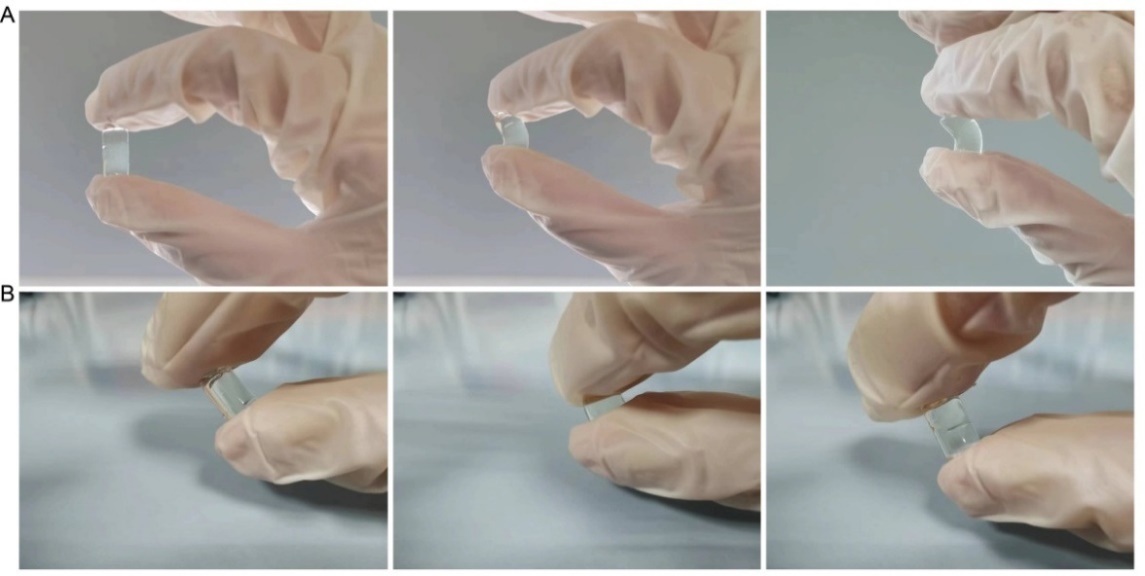


**Figure S2.** Compression and bending resistance tests of GelMA hydrogel.


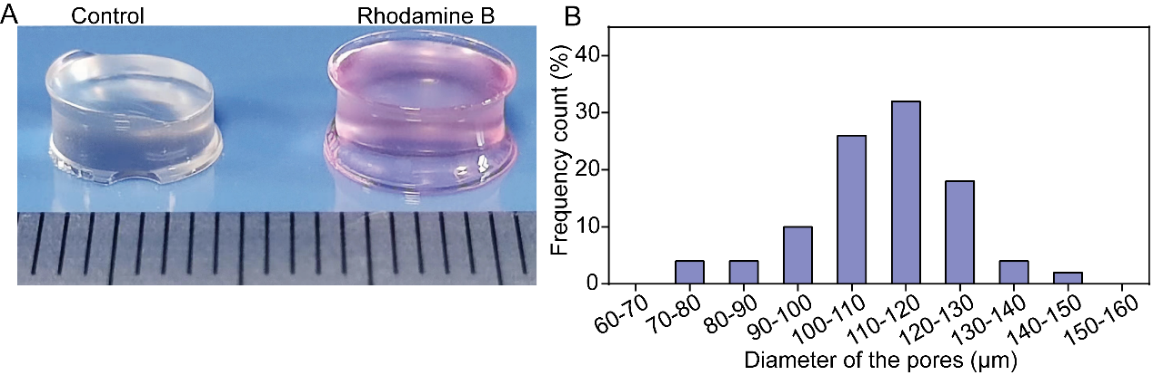


**Figure S3.** (A) The images of GelMA + PEO hydrogel and rhodamine B-labelled GelMA + PEO hydrogel. (B) Pore size distribution of GelMA+PEO hydrogel.


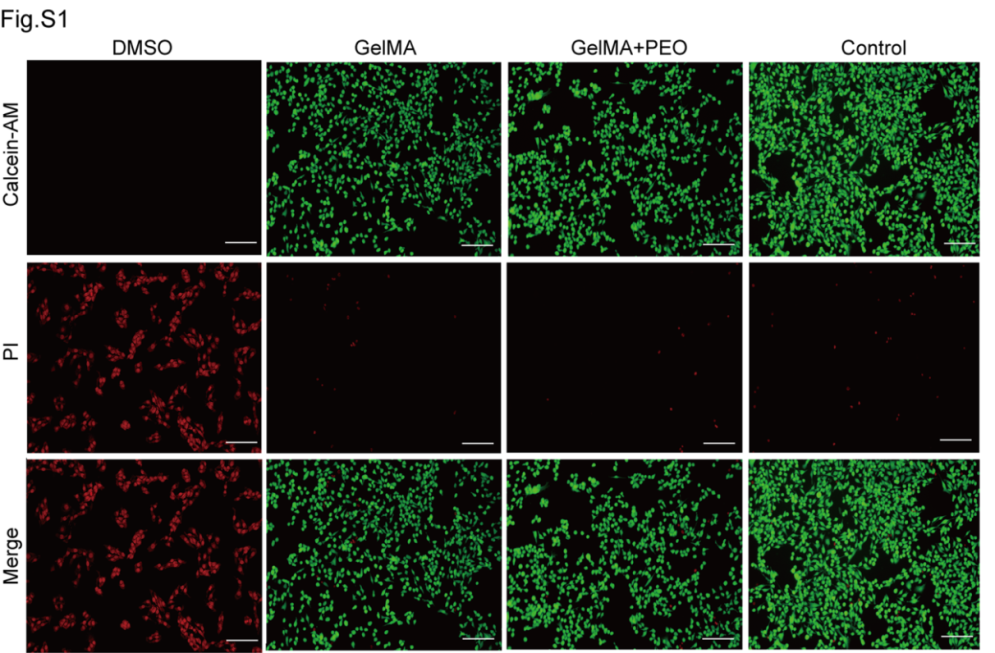


**Figure S4.** Viability determination of B16 cells. The Calcein-AM, PI staining and merge images of B16 cells cultured respectively with DMSO and the extraction solution of GelMA and GelMA + PEO hydrogel. (Green remarked live cells, red remarked dead cells). Bar = 50 μm.


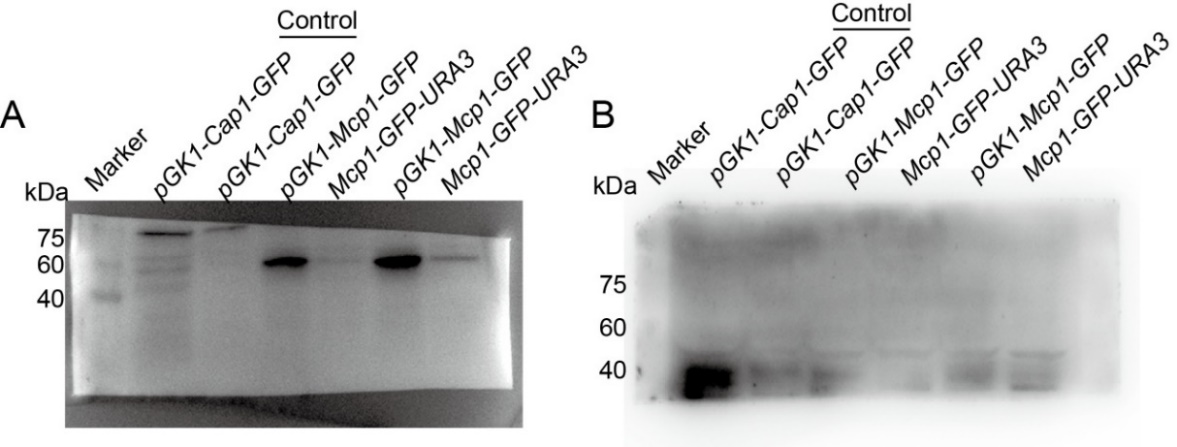


**Figure S5.** (A) A full scan of Figure 3G (Mcp1-GFP) entire original gel; (B) A full scan of Figure 3G (Tubulin) entire original gel.


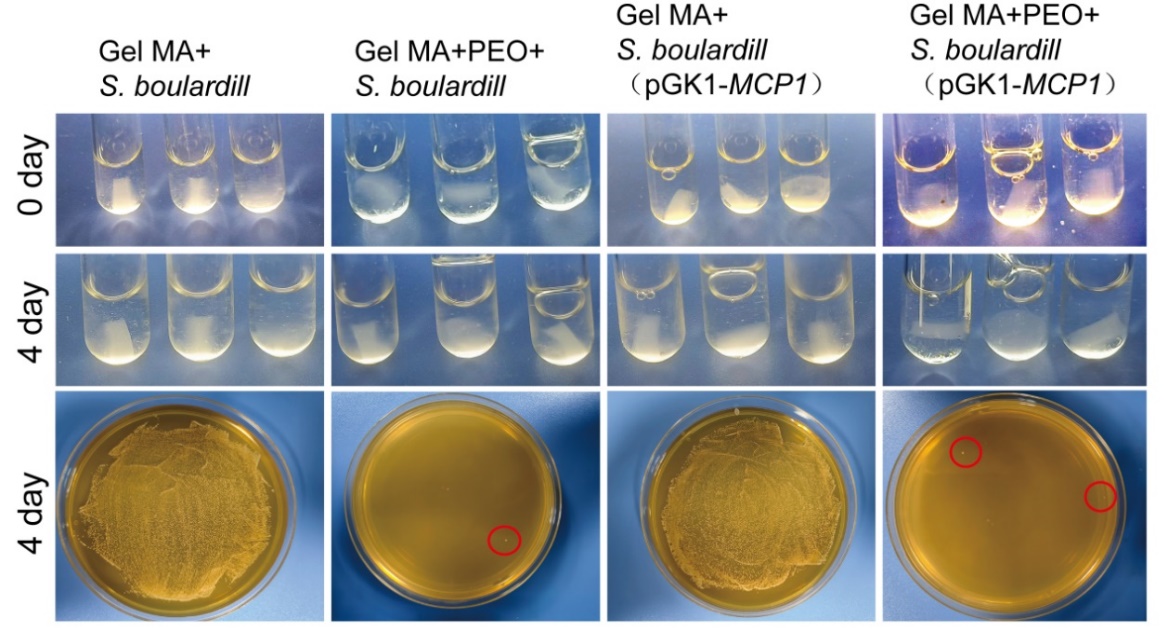


**Figure S6.** Leaching test of *S. boulardii* encapsulated in coating (A) The images of bio-coatings containing *S. boulardii* and transgenic *S. bourlardii* were immersed in physiological saline (0.9% NaCl) for 4 days, respectively. (B) Photos of *S. boulardii* and transgenic *S. bourlardii* colonies on agar broth plates separated from the soaking solution with different bio-coating.


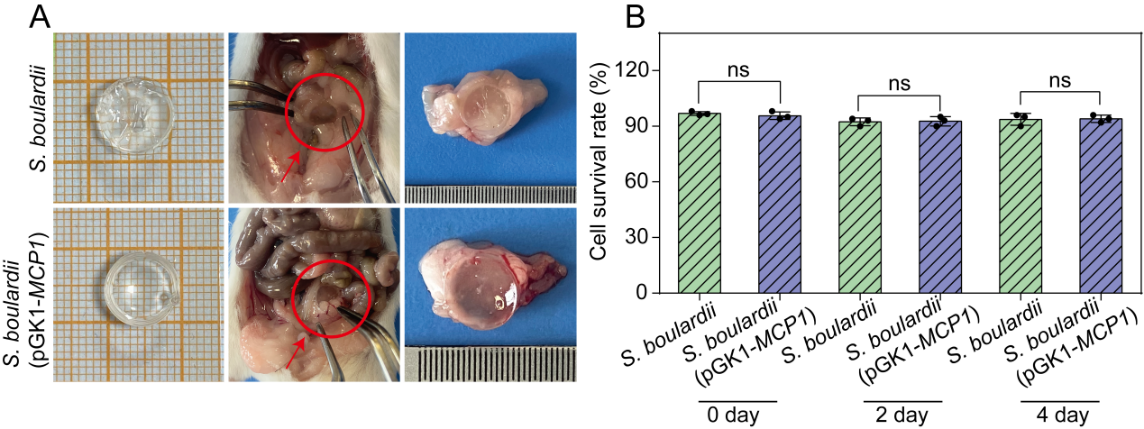


**Figure S7.** In vivo implanted studies (A) Photos of *S. boulardii* hydrogels in vitro and in vivo, and the attachment tissue sections with different hydrogels derived from mice. (B) The cell survival rate of *S. boulardii* and transgenic S. *boulardii* encapsulated in hydrogels after implanted in vivo for 4 days. Statistical significance was defined based on the different *p*-values: **p*<0.05, ***p*<0.01, and ****p*<0.001.


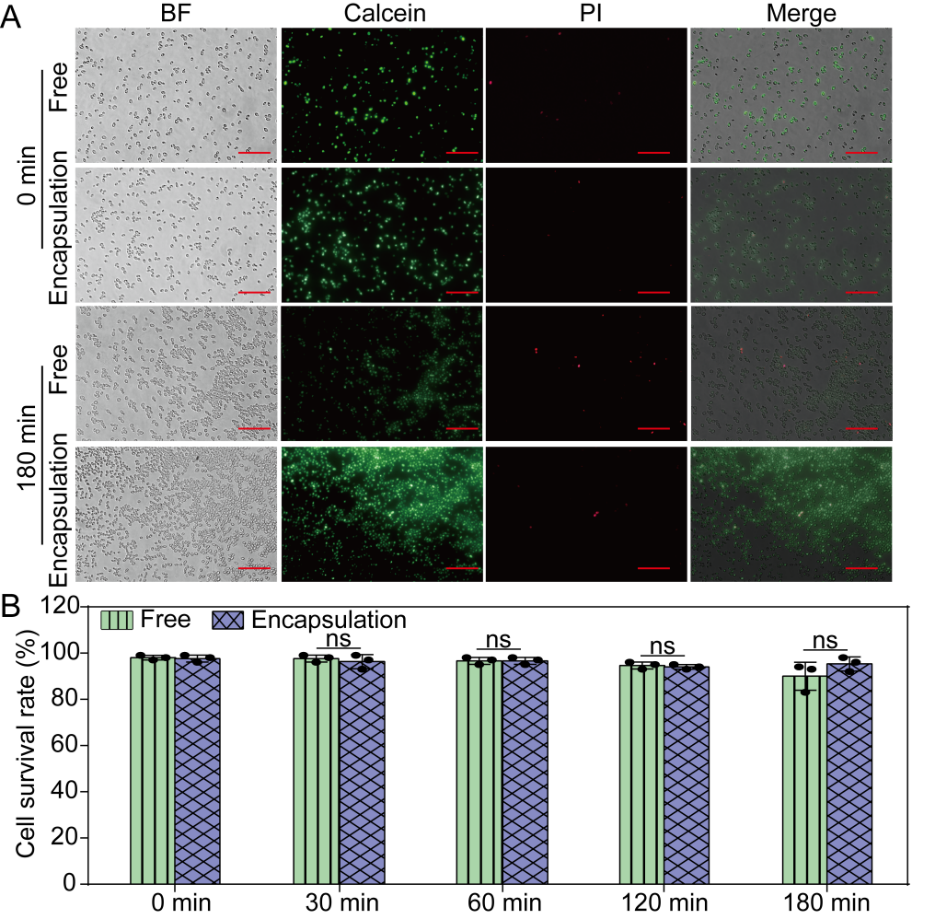


**Figure S8.** (A) Calcein-AM/PI staining of *S. boulardii* in bioactive materials under condition of UV at 180 min. (Green remarked live cells, red remarked dead cells) Bar = 50 μm. (B) The cells survival rate of *S. boulardii* in bioactive materials under condition of UV at 30, 60, 120, and 180 min. Statistical significance was defined based on the different *p*-values: **p*<0.05, ***p*<0.01, and ****p*<0.001.


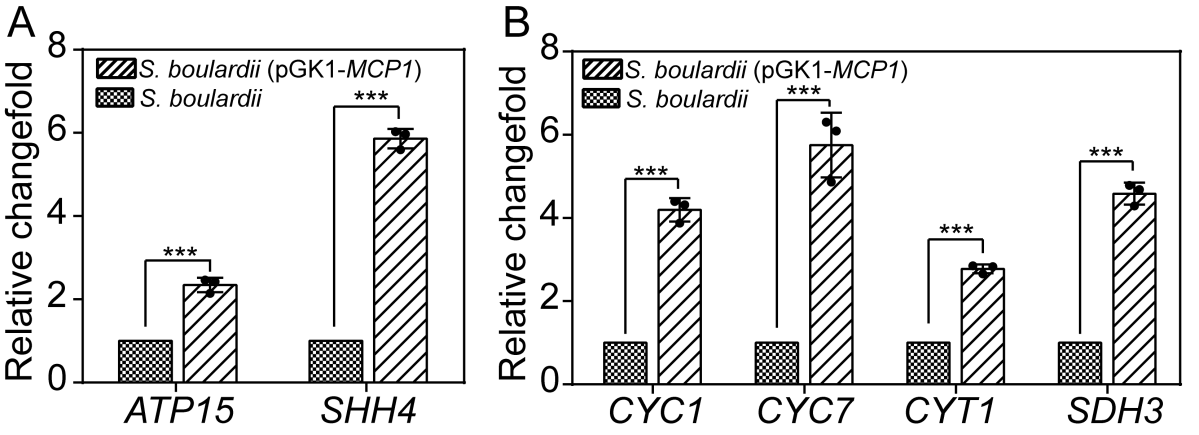


**Figure S9.** Overexpression of *MCP1* enhances mitochondrial function of *S. boulardii* in bioactive materials after Co-incubation with *C. albicans*. (A) The expression levels of mitochondrial related genes *ATP15* and *SHH4* of *S. boulardi* revealed by RT-PCR. Expression levels of electron transport chain related genes *CYC1*, *CYC7*, *CYT1* and *SDH3* revealed by RT-PCR. *ACT1* was used as the normalization gene. Statistical significance was defined based on the different *p*-values: **p*<0.05, ***p*<0.01, and ****p*<0.001.
